# Optimizing Immunostaining of Enamel Matrix: Application of Sudan Black B and Minimization of False Positives from Normal Sera and IgGs

**DOI:** 10.3389/fphys.2017.00239

**Published:** 2017-04-25

**Authors:** Xu Yang, Alexander J. Vidunas, Elia Beniash

**Affiliations:** ^1^Department of Oral Biology, School of Dental Medicine, University of PittsburghPittsburgh, PA, USA; ^2^Department of Bioengineering, Center for Craniofacial Regeneration, Swanson School of Engineering, McGowan Institute for Regenerative Medicine, University of PittsburghPittsburgh, PA, USA

**Keywords:** amelogenesis, enamel, immunofluorescence microscopy, false positive, Sudan Black B

## Abstract

Non-specific fluorescence from demineralized enamel matrix can significantly compromise the immunofluorescence studies and lead to false positives. Our goal was to assess degrees of non-specific binding under different conditions and try to optimize procedures for immunofluorescence studies of forming enamel. Firstly, we compared two methods for background fluorescence elimination, i.e., sodium borohydride and Sudan Black B treatments. The results demonstrated that Sudan Black B is far superior to sodium borohydride in reducing the background fluorescence in dental tissues. We also studied the extent of non-specific binding of normal sera and purified polyclonal immunoglobulins (IgG) from five mammalian species, guinea pig, rat, rabbit, goat, and sheep, over a broad range of dilutions. For all sera tested fluorescence signals increased exponentially from 1:1000 to 1:100. Interestingly, the non-specific binding of sera from rodent species was below that of positive control in the whole range of dilutions. In contrast, incubation with sera from 3 non-rodent species produced much higher signals which surpassed the positive control signal at 1:250~1:500 dilution range. Most of the IgGs didn't show significant non-specific binding within 0.25–5 μg/ml range, except rabbit IgG which demonstrated extremely high affinity to the enamel matrix even at concentrations as low as 1 μg/ml. Further, studies confirmed that Fab fragments of purified normal rabbit IgG, not conserved Fc fragments, were involved in the interactions. Our observations suggest this high affinity is associated with the antigen binding sites of rabbit IgG. We anticipate that our results will help enamel researchers to optimize and standardize their immunochemical procedures.

## Introduction

Although mature enamel is the hardest tissue of the human body which primarily comprises carbonated apatite with <1% w/w organics, it starts as a tissue with ~30% organic matrix by weight (Margolis et al., 2006). Unlike other mineralized tissues, such as bone and dentin, which contain roughly 30% of collagenous matrix, most of the enamel organic matrix is degraded during the maturation stage (Simmer and Hu, 2002). Studies of enamel secretion and maturation are key for our understanding of enamel mineralization strategies. These studies can provide valuable information about enamel formation in norm and disease and an inspiration for design of novel nanostructured hierarchical materials.

Immunofluorescence is a powerful tool, which can provide wealth of information regarding structural and functional properties of biological samples. One of the perennial problems researchers face when using this technique are false positives due to autofluorescence or non-specific antibody binding which, if not taken into account can lead to wrong conclusions (Baschong et al., 2001; True, 2008; Tan et al., 2012). Although no systematic studies of autofluorescence or non-specific staining of enamel were published, enamel researchers are generally aware of these issues and interpret immunofluorescence studies of amelogenesis with caution.

Sudan Black B (SBB) is widely used to eliminate autofluorescence in histology studies, although exact mechanisms of its action are unknown. It was shown to dramatically reduce background signals not only in biological tissues (Romijn et al., 1999; Viegas et al., 2007; Oliveira et al., 2010; Nakata et al., 2011; Sun et al., 2011; Yang and Honaramooz, 2012; Neo et al., 2015; Erben et al., 2016; Kajimura et al., 2016), such as lymph node, thymus, liver, kidney, pancreas, testis, brain, and silk, but also in synthesized polymers (Jaafar et al., 2011). Another chemical which is widely used to reduce autofluorescence from aldehyde fixed samples is NaBH_4_ (Clancy and Cauller, 1998; Davis et al., 2014). In this study we compared two methods of reducing non-specific staining in decalcified mouse enamel matrix. We also investigated interactions between the enamel matrix and normal sera or polyclonal immunoglobulins (IgGs) from a number of mammalian species. These studies were conducted over a broad range of dilutions typically used in the immunochemistry studies. We hope that the information presented in this paper will help other researchers to better design and interpret the immunofluorescence studies of dental tissues.

## Materials and methods

### Sample preparation

Four weeks old wild type C56BL/6J mice were sacrificed according to an approved protocol. Mandibles were dissected out immediately and fixed with 4% paraformaldehyde in PBS for 24 h at 4°C. Fixed mandibles were kept in 8% EDTA solution for 1 week, and the solution was changed every other day. De-mineralized mandibles were then embedded in paraffin blocks and 8 μm sections were prepared using a Leica RM2245 (Leica Biosystems, Nussloch, Germany). The sectioning was conducted in the coronal plane at the location of the first molar. Serial sections from three different animals were used. For fluorescence blocking study, additional sectioning was conducted in the sagittal plane.

### Antigen retrieval and blocking procedures

Sections were de-paraffinized and treated with trypsin-EDTA (Sigma, T4049) for 10 min at 37°C for antigen retrieval, then blocked with 10% Donkey serum, 2.5% BSA (Jackson Immunoresearch, 001-000-161), 0.1% Triton X-100 (Sigma, T9284), 0.15% glycine (Sigma, 410225), 0.25% casein (Fisher, BP-337) and 0.1% gelatin (Sigma, G7765) in Tris-buffer solution plus 0.05%Tween-20 (TBST) for 1 h.

### Non-specific fluorescence blocking

In the experiment aimed at comparing the effects of different non-specific fluorescence blockers serial sections were grouped by three treatments. One group was treated with 1 mg/ml of freshly made sodium borohydride (NaBH_4_) solution before the blocking step. Sections were incubated 10 min X 3 with NaBH_4_ and washed three times with TBST. The sections were incubated with or without secondary antibodies (1:500, see Table 1) for an hour at room temperature, followed by TBST washing and DAPI staining. Another group was treated with 1.5% Sudan Black B (SBB) in 70% ethanol (SBB, filtered before use) after secondary antibody (1:500) incubation or in ½ blocking buffer only for 1 h. The sections were washed with TBST four times, then incubated with SBB for 20 min, washed with TBST and stained with DAPI. Untreated sections with or without secondary antibodies incubation were used as controls.

**Table 1:** **The product information for antibodies used in the study**.

	**Donkey**	**Goat**	**Guinea pig**	**Rabbit**	**Rat**	**Sheep**
Sera	017-000-121^*^	005-000-121^*^	006-000-001^*^	011-000-001^*^	012-000-001^*^	013-000-001^*^
IgG		sc-2028^#^	sc-2711^#^	sc-2027^#^	sc-2026^#^	sc-2717^#^
2nd Ab		705-165-147^*^	706-165-148^*^	711-165-152^*^ 711-545-152^*^	712-165-150^*^	713-165-147^*^
	**Thermo fisher**	**Millipore**	**Jackson immuno**	**DSHB**	**Fab**	**Fc**
Other	026102	NI01	011-000-003	CPTC-CALR-1-s	011-000-007	011-000-008
Rb IgG						

* *Sera were purchased Jackson Immunoreasearch Inc. from PA, USA*.

# *IgGs were purchased from Santa Cruz Biotechnology, Inc. from CA, USA*.

### Non-specific binding studies

In the study of non-specific interactions of sera and IgGs with dental tissues, sections incubated overnight in the 2 times diluted blocking buffer at 4°C (see the Antigen retrieval and blocking procedures section for the blocking buffer composition). Some of these sections were used as negative control. For positive controls, after blocking sections were incubated with affinity purified ameloblastin (Santa Cruz, sc-50534) or enamelin (Santa Cruz, sc-33107) antibodies at concentration of 1 μg/ml overnight at 4°C. Serial sections from 2 incisors were incubated overnight at 4°C with sera from different species (see Table 1) at 1:1000, 1:500, 1:250 and 1:100 series of dilutions. Other serial sections were incubated overnight at 4°C with IgGs obtained from different species at concentrations of 0.25, 1, 2.5, and 5 μg/ml (Table 1). In an additional set of experiments we tested normal rabbit IgGs from 4 different manufacturers (see Table 1), Fab fragment and Fc fragment and rabbit anti-calreticulin peptide 1 (CP1) monoclonal IgG (Developmental Studies Hybridoma Bank, CPTC-CALR-1-s). In this set of experiment a concentration of 2 μg/ml was used. The sections were washed with TBST 5 min for six times and incubated with secondary antibodies (see Table 1) at 1:500 dilution in ½ blocking buffer for an hour. SBB was used to eliminate the background and DAPI (0.2 μg/ml for 5 min) was used as counter stain. Importantly, for all fluorescence studies adjacent serial sections were used to minimize intra and intergroup variability.

All sections were scanned using Nikon A1 confocal microscope (Nikon Instruments, Melville, NY) at Center for Biologic Imaging (CBI) of University of Pittsburgh. Sections from same block were scanned under the same imaging conditions. The images were captured in 16 bit RGB mode and stored in the native nd2 or tiff file format.

### Data analysis

All images were analyzed in NIS-Elements AR software (Nikon Instruments, Melville, NY) or in imageJ image analysis package (Bethesda, MD). In general, similar areas of interest (at least 1,000 μm^2^) were selected and average signal intensity values were measured. The data was plotted using Origin 2016 graphing software (Origin Labs, Waltham, MA).

## Results

### Comparison of the NABH_4_ and SBB for reduction of auto-fluorescence

**Figure 1:**
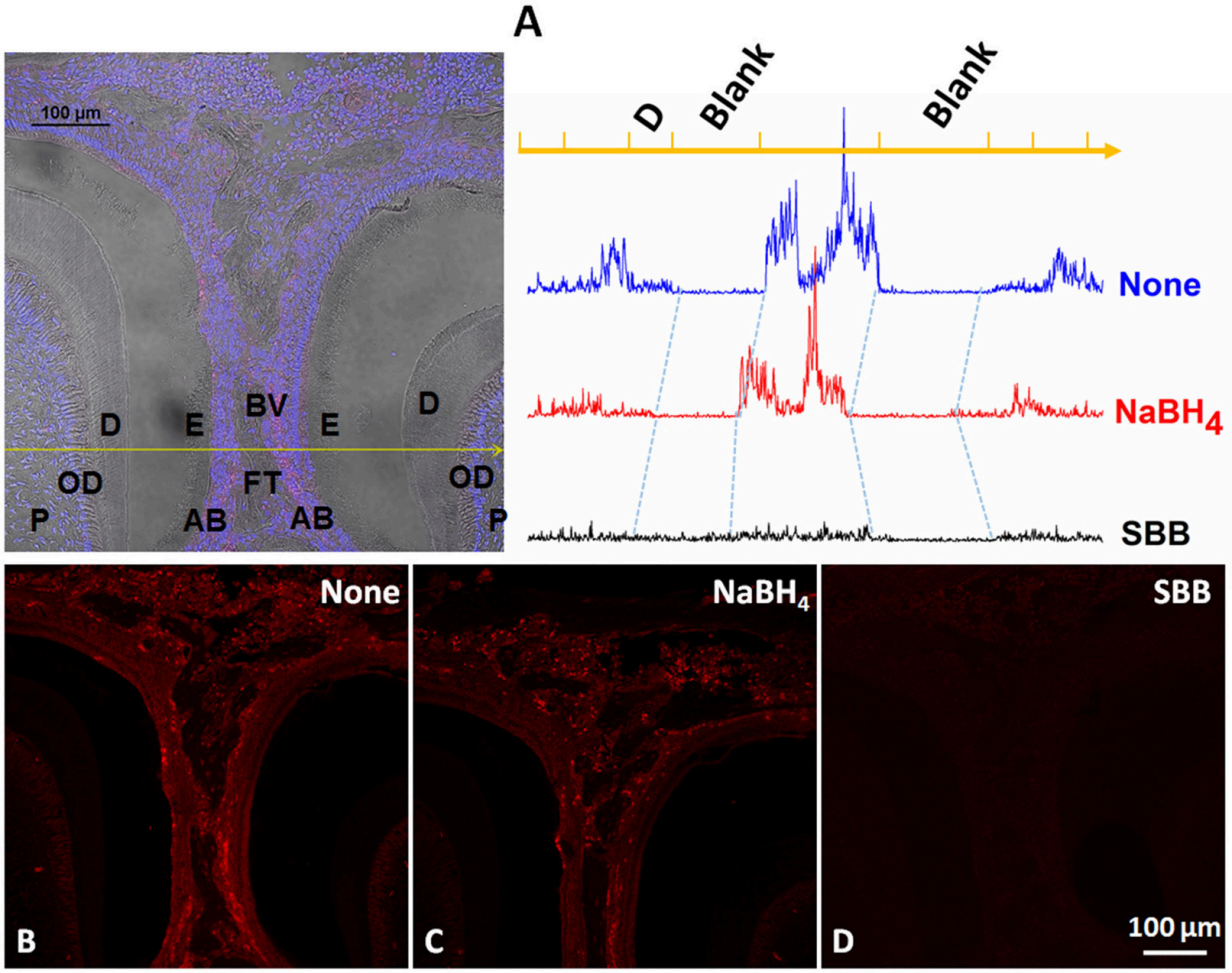
**Comparison of the autofluorescence blocking by NaBH_**4**_ and SBB in sections of mouse mandibles**. **(A)** Left. Multi-channel image, containing bright field, DAPI and TRITC channels, of a section including parts of 1st and 2nd molar. Yellow line identifies the location at which the fluorescence profiles on the right were obtained in the adjacent sections shown in **(B,C)** and Right, **(D)**, fluorescence profile on the top is from th non-treated section **(B)**; middle profile is from the NaBH_4_ treated section **(C)** and the bottom profile is from the SBB treated section **(D)**. P, pulp; OD, odontoblast; D, dentin; E, enamel; AB, ameloblast; BV, blood vessel; FT, fibrous tissue.

Strong non-specific signal was observed in the control group (Figures 1A,B). It was strongest in fibrous tissue and blood vessels while lower in odontoblasts, ameloblasts, enamel and dentin. Treatment with NaBH_4_ lead to a minimal decrease in fluorescence signal compared to the control (Figures 1A,C), while incubation in SBB greatly reduced staining of all tissues in the sections (Figures 1A,D). No obvious differences were found after incubation with secondary antibody in each group.

### Reactivity of sera from different species with the enamel matrix

**Figure 2:**
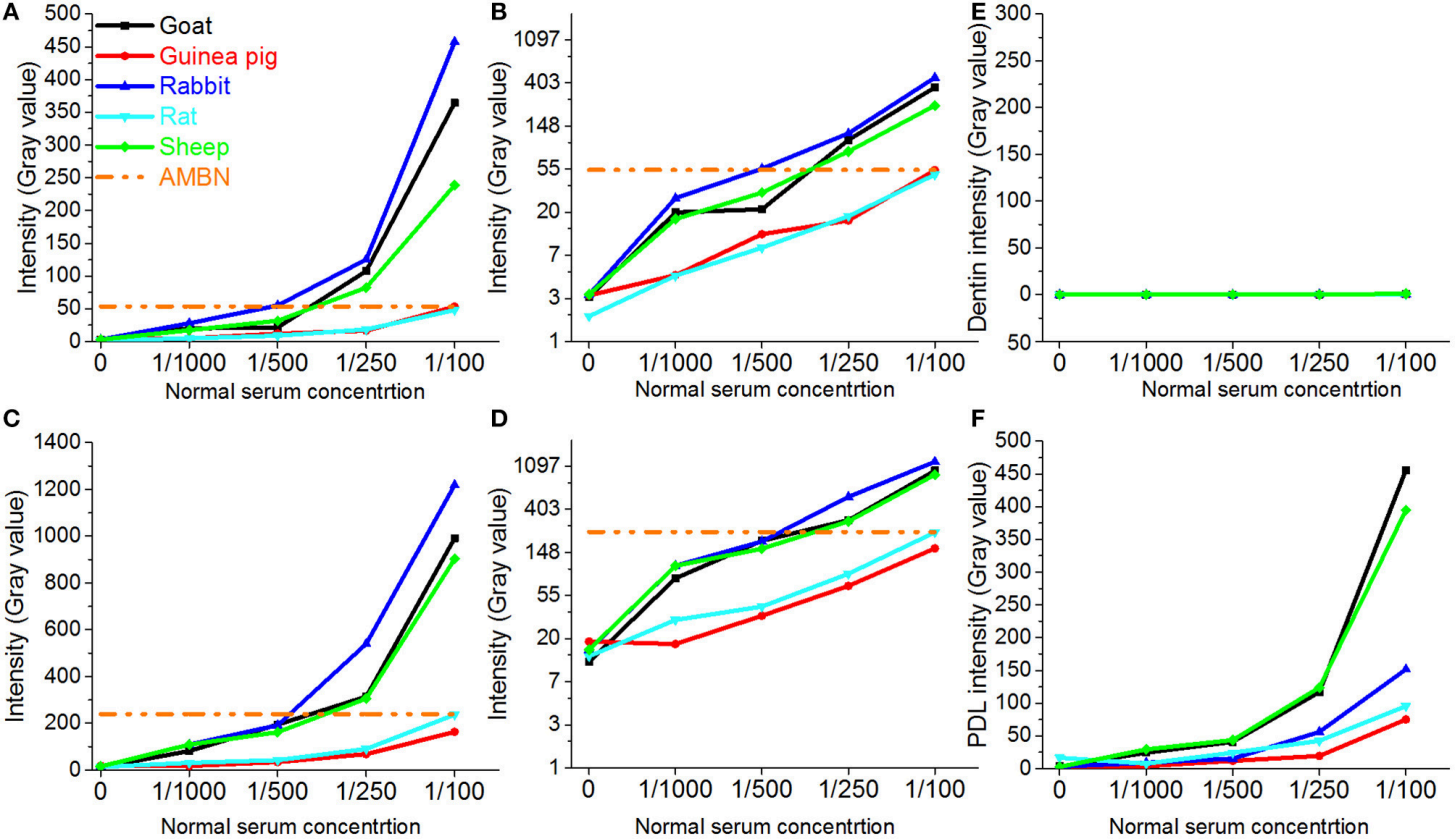
**Changes in fluorescence intensities of different dental tissues incubated with sera from different species. (A)** Fluorescence intensity profiles of enamel matrix from incisor 1; **(B)** data in **A** presented on the natural logarithmic scale; **(C)** Fluorescence intensity profiles of enamel matrix from incisor 2; **(D)** data in **(C)** presented on the natural logarithmic scale; **(E)** Fluorescence intensity profiles of dentin matrix from incisor 1; **(F)** Fluorescence intensity profiles of periodontium from incisor 1.

**Figure 3:**
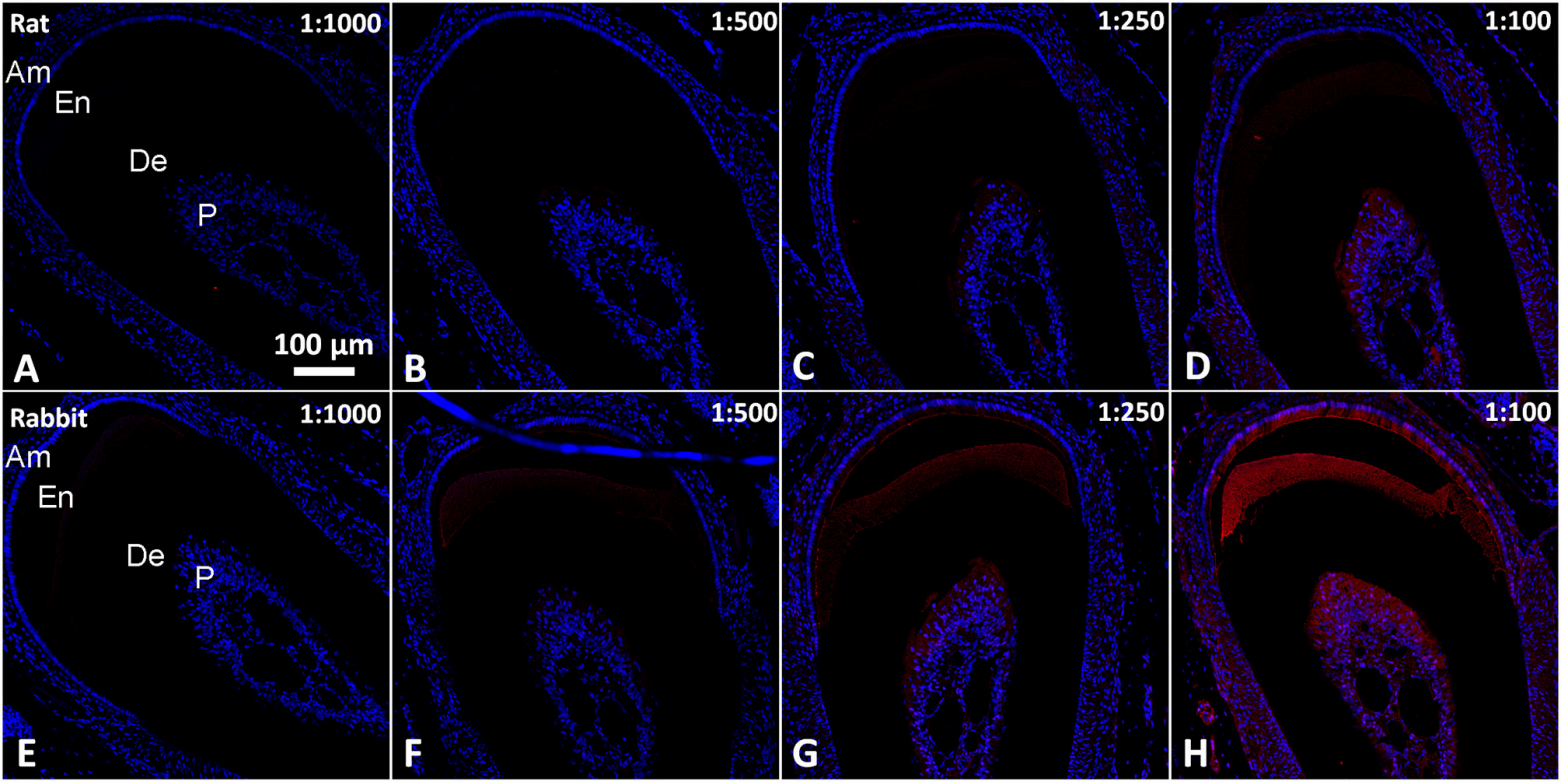
**Epifluorescence micrographs from serial coronal sections of mouse incisor incubated with normal sera in in the 1/1000–1/100 range of dilutions**. **(A–D)** Rat serum. **(E–H)** Rabbit serum. All images were taken under the same magnification. Am, ameloblasts and stratum intrermedium; De, dentin; En, enamel; P, Pulp.

Fluorescence at the 1:1000 dilution of all sera was at the level of the background signal (Figures 2A,C). The fluorescence increased exponentially with the increase of sera concentrations. Plotting the data on a natural logarithm (Ln) scale lead to linearization of the curve, which confirmed the exponential relationships between the sera dilution and the fluorescence intensity (Figures 2B,D). Interestingly, the fluorescence signals in experiments with sera from rodent species, rat and guinea pig, increased ~10 times from 0 to 1/100 dilution, while fluorescence from the samples treated with sera from other mammalian orders, i.e., rabbit, goat, and sheep increased up to 100 times over the same range of dilutions (Figure 3). Importantly, the non-specific signal from the samples treated with rodent sera reached the signal intensity level of the positive control at 1/100 dilution, while for the non-rodent sera this level of intensity was reached at around 1/500. Adjacent periodontal ligament (PDL) tissue showed non-specific fluorescence levels similar to forming enamel while signal from dentin tissue was always at the background level (Figures 2E,F).

### Reactivity of IgGs from different species with enamel matrix

**Figure 4:**
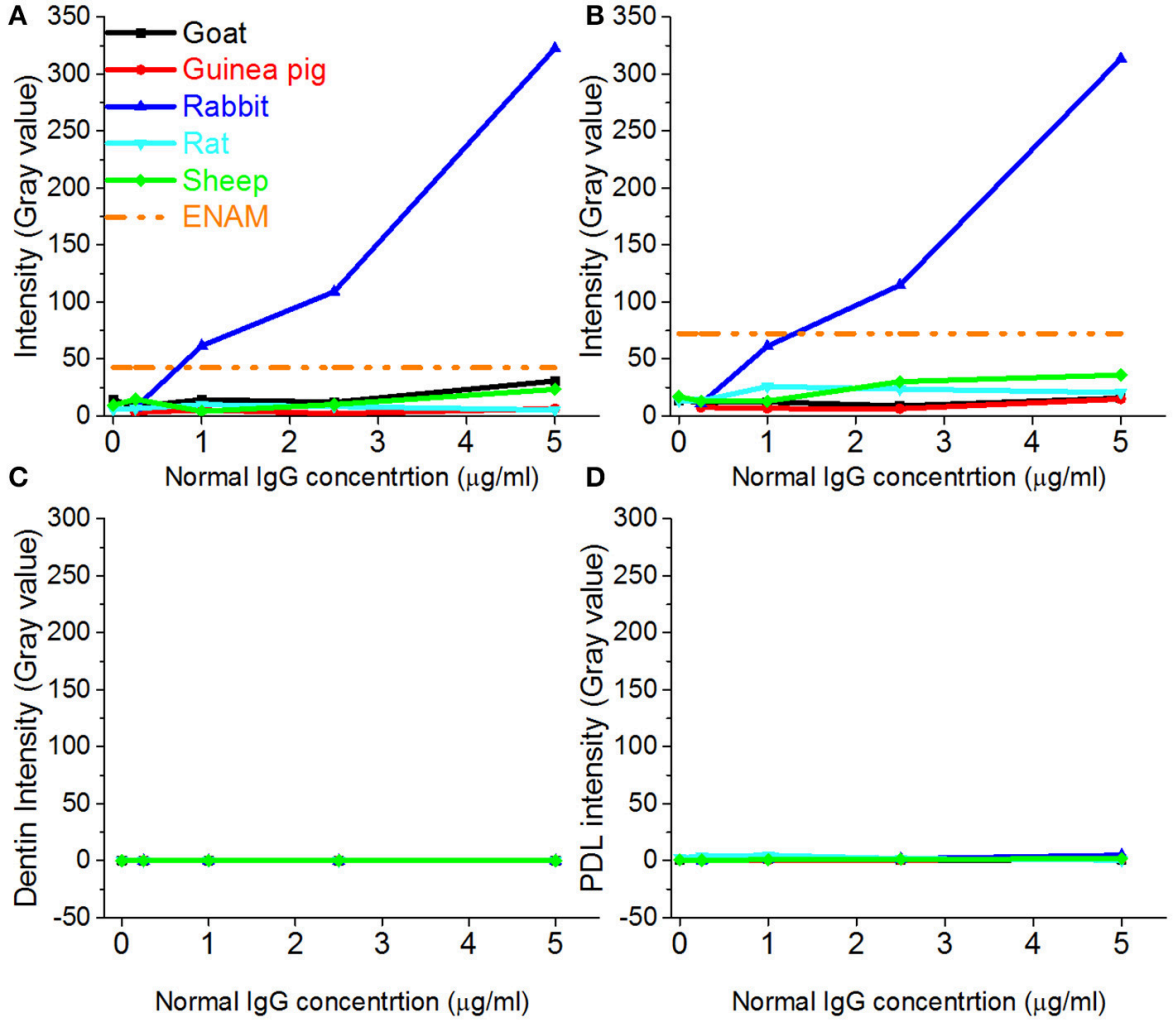
**Changes in fluorescence intensities of different dental tissues incubated with IgGs from different species**. Fluorescence intensity profiles of enamel matrix from incisor 1 **(A)**, incisor 2 **(B)**, dentin from incisor 1 **(C)** and periodontium from incisor 1 **(D)**.

**Figure 5:**
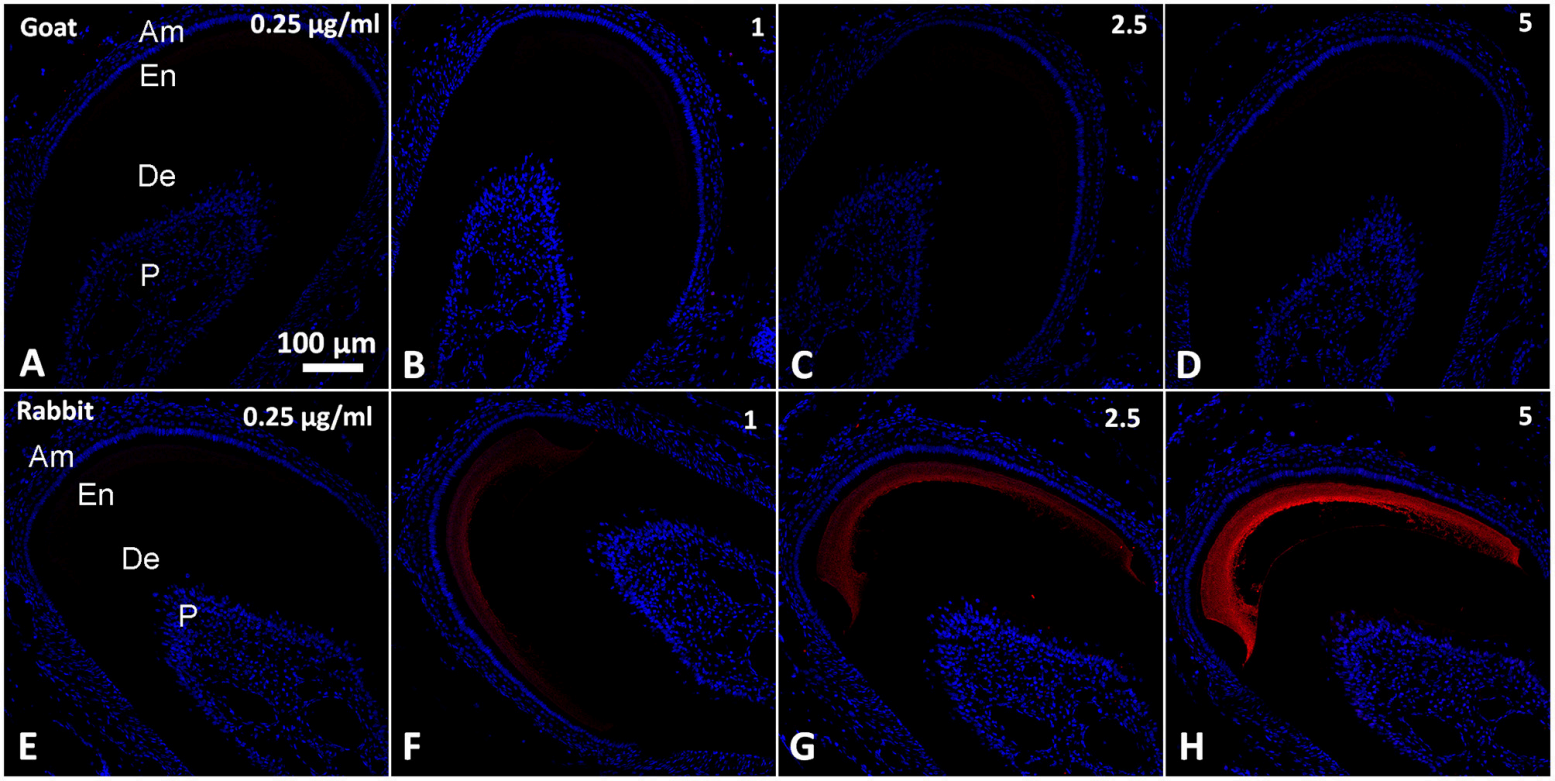
**Epifluorescence micrographs from serial coronal sections of mouse incisors incubated with normal IgGs in a range of concentrations from 1 to 5 μg/mL. (A–D)** Goat IgG; **(E–H)** Rabbit IgG. All images were taken under the same magnification. Am, ameloblasts and stratum intrermedium; De, dentin; En, enamel; P, Pulp.

**Figure 6:**
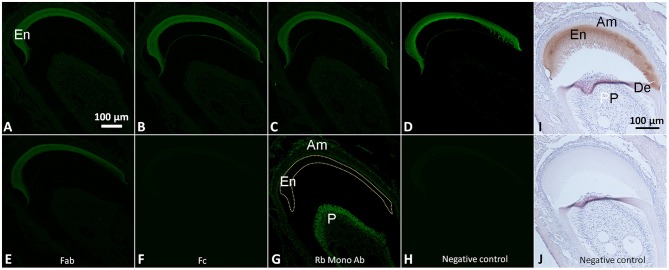
**Immunofluorescence images of serial coronal sections of mouse incisors incubated with normal IgGs from Thermo Fisher (A)**, Jackson Immunoresearch **(B)**, Millipore **(C)** and Santa Cruz **(D)**, Fab **(E)** and Fc **(F)** fragments and the monoclonal rabbit antibody against CP1 **(H)**. Note that in **(H)** the staining is only associated with cells, not the enamel matrix. **(I,J)** show bright field micrographs of the immunohistochemistry staining with normal rabbit IgGs and the negative control, respectively. Note that the fluorescence signal is present in enamel matrix (En) after incubation with rabbit IgGs and the Fab fragment while no obvious signals are present after Fc incubation and in the negative control. **(A–H)** were acquired under the same magnification; **(I,J)** were acquired under the same magnification. All incubations were conducted at 2 μg/mL concentrations of antibodies. Abbreviations are the same as in Figure 1. Am, ameloblasts and stratum intrermedium; De, dentin; En, enamel; P, Pulp.

In contrast to sera, most of the IgGs exhibited low level of fluorescence over the range from 0 to 5 μg/ml (Figures 4A,B), well below the intensity levels of positive controls, with the exception of rabbit IgG which strongly reacted with enamel matrix (Figures 4A,B blue line, Figures 5E–H). The fluorescence intensity of the rabbit IgG grew linearly with the increase in concentration. It reached the fluorescence levels of the positive control around 1 μg/mL and was 50–100 times higher than the baseline fluorescence at the maximum concentration of 5 μg/ml. Adjacent tissues such as PDL and dentin didn't show an affinity to rabbit IgG and their fluorescence remained low in the range of the concentrations tested (Figures 4C,D, 5). There were no major differences between rabbit IgGs from different manufacturers tested in the study (Figures 6A–D). The same phenomenon was also observed in routine immunohistochemistry staining (Figures 6I,J).

### Reactivity of rabbit monoclonal antibodies and IgG fragments with enamel matrix

Rabbit monoclonal antibody against CP1, a ER marker, did not interact with the enamel matrix, while adjacent cells demonstrated a strong signal (Figure 6G). At the same time rabbit Fab fragments showed similar binding to enamel matrix as rabbit IgG whole molecule (Figure 6E). When the sections were incubated with Fc fragment only, no signal was detected (Figure 6F). No signal was detected in the negative control (Figure 6H).

## Discussion

Our results clearly demonstrate an excellent ability of SBB to block the non-specific signal from the enamel matrix and other tissues. This was in a good agreement with the results of autofluorescence quenching by SBB in other tissues (Viegas et al., 2007; Davis et al., 2014; Erben et al., 2016). In fact, fluorescence of almost all the tissues in SBB treated sections were reduced to a level close to the background signal. At the same time NaBH_4_ had little or no effect on the non-specific fluorescence, suggesting that this method is not appropriate for dental tissues. A potential explanation of the low effectiveness of NaBH_4_ might lie in the fact that after fixation the samples in our study underwent a decalcification step which involves prolonged incubation in EDTA, which might reduce the levels of aldehyde crosslinks, resulting in reduced aldehyde-related autofluorescence.

In the course of our immunochemical studies we noticed repeatedly a very high background signal in enamel matrix treated with normal rabbit IgG as an isotype control. To systematically investigate this phenomenon, we tested the levels of non-specific binding to enamel matrix of normal sera and IgGs from several commonly used mammalian species. Our results demonstrate high levels of fluorescence in the mouse enamel matrix treated with sera from the non-rodent species. The fluorescence intensity for sera from 3 non-rodent species was at the level of the positive control at the dilutions of 1/500 or less. Remarkably, sera from two rodent species showed much lower degree of non-specific binding, perhaps due to the evolutionary proximity between the host and target species. Our results indicate that caution should be taken when using goat, sheep and rabbit sera at the dilutions around 1:500 or less, while for rat and guinea pig sera using dilutions lower than 1:100 is not recommended (Figures 2A,C). It is important to note that dentin tissue has a very low level of non-specific binding for all sera, suggesting that it cannot be used as an internal control in immunofluorescence studies of forming enamel.

In contrast to the results of the experiments using whole sera, most affinity purified IgGs showed low affinity to the mouse enamel matrix across concentrations ranging from 0.25 to 5 μg/ml, and the fluorescence levels of the IgG treated samples were close to the background levels over this range. The only exception was rabbit IgG which showed very high affinity to the enamel matrix. To exclude the possibility that this strong binding was limited to IgGs form Santa Cruz, rabbit IgGs from three other manufacturers were also tested, and all of them showed high levels of binding. In order to understand which portion of the IgG interacts with enamel matrix, we also examined Fc (conserved) and Fab (variable) fragments from IgG purified from normal rabbit serum. Our results clearly demonstrated that the variable Fab fragment strongly interacts with enamel matrix, while the conserved Fc portion did not bind to the enamel matrix. At the same time, monoclonal anti-CP1 rabbit IgG didn't show any affinity to the enamel matrix. Together these data suggest that the high affinity of purified IgGs and Fab fragments to the enamel matrix is not an intrinsic property of any rabbit immunoglobulin but is specific to the certain antigen binding site. This observation was quite unexpected and very intriguing. Our recent study showed the existence of keratins in enamel matrix (Duverger et al., 2014), and based on the personal communication by Dr. Maria Morasso (NIH, Bethesda, MD), even normal rabbit serum might react with keratins because rabbits often get wounded while scratching which presents an opportunity for skin and hair keratins to end up in the blood stream. However, at this time we can only surmise what are the reasons for such high affinity of rabbit IgG to the enamel matrix. More studies will be necessary to understand the mechanisms behind the phenomenon.

## Conclusion

We conducted a number of experiments aimed at optimization of the immunofluorescence procedure for the extracellular matrix of forming enamel. Specifically, we showed that treatment of sections with SBB leads to significant reduction in autofluorescence of all dental tissues. We also determined the ranges of sera dilution and concentrations of IgG which can be used to minimize false positives, suggesting that proper isotype controls will be necessary when working beyond this range. Our observations indicate that rabbit sera and IgG have a very high affinity to the enamel matrix, and this high affinity is associated with the antigen binding sites.

## Ethics statement

This study was carried out in accordance with the recommendations of IACUC of the University of Pittsburgh. The protocol was approved by the IACUC of the University of Pittsburgh.

## Author contributions

XY and EB participated in the study design, data analysis and manuscript writing. XY and AV conducted the experiments.

### Conflict of interest statement

The authors declare that the research was conducted in the absence of any commercial or financial relationships that could be construed as a potential conflict of interest.
